# Supplementary material for: MicroRNA-708 emerges as a potential candidate to target undruggable NRAS
Source: PLoS One. 2023 Apr 21;18(4):e0284744. doi: 10.1371/journal.pone.0284744 (PMC10120925; doi:10.1371/journal.pone.0284744)
Supplement: S1 File — (PDF) [file pone.0284744.s001.pdf]

Supplementary Information

MicroRNA-708 emerges as a potential candidate to target undruggable NRAS

Jia Meng Pang *et al.*,

Corresponding author: Kai-Ti Lin (ktlin@life.nthu.edu.tw)

The PDF file includes:

Figure S1. MicroRNA-708 is predicted to target *NRAS* gene through TargetScan prediction

Figure S2. Level of miR-708 after transfection

Figure S3. MicroRNA-708 exerts tumor suppressive function in H1299 and THP-1 cells.

Figure S4. Uncropped images underlying of all blots.

Table S1. Primer and small RNA sequences.

Other Supplementary Material for this manuscript includes the following:

Table S2. Clinical information of SKCM, LUAD, and LAML patients and miR-708 expression data retrieved from the TCGA cohort.

A

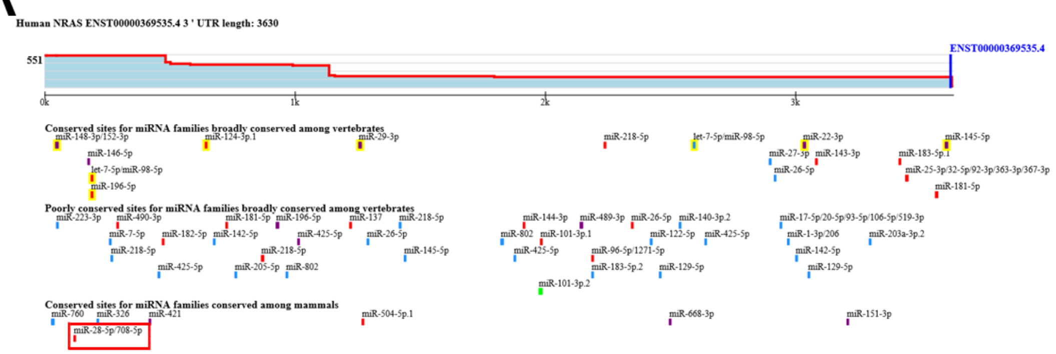

B

	Predicted consequential pairing of target region (top) and miRNA (bottom)	Site type	Context++ score	Context++ score percentile	Weighted context++ score	Conserved branch length	P_{CT}
Position 116-122 of NRAS 3' UTR<br>hsa-miR-708-5p	5' ...UCAGUCUCACAGAGAAGCUCCUG...<br>||||||| 3' GGGUCGAUCUAACAUUCGAGGAA	7mer-m8	-0.18	89	-0.18	3.573	N/A

Figure S1. MicroRNA-708 is predicted to target *NRAS* gene through TargetScan prediction (A) The TargetScan page for the potential microRNA targeting on the 3' UTR of the *NRAS* gene. The position of targeting sites by miR-708 is highlighted by the red box (B) The alignment of the predicted pairing between the miRNA-708 and its sites on *NRAS* 3' UTR, showing the position, site type, context++ score, context++ score percentile, weighted context++ score, branch-length score, and PCT score.

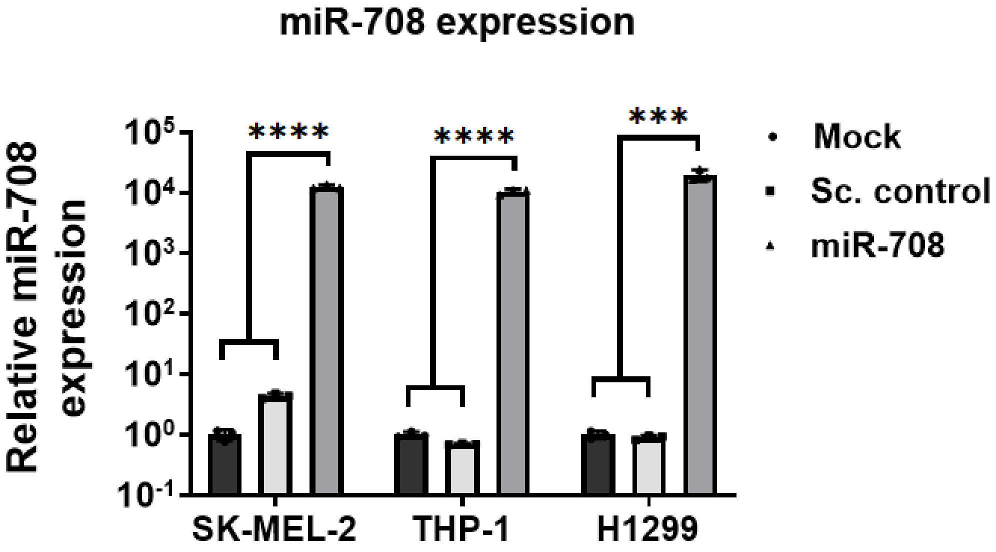

Figure S2. Level of miR-708 after transfection. SK-MEL-2, THP-1, and H1299 cells were transfected with pre-miR-708 for 48 h then subjected to RT-qPCR analysis and examined miR-708 level to confirm transfection efficiency. Histogram showing means ± SD (n=3 technical replicates). One-way ANOVA followed by Tukey post-hoc test was used for the statistical test (***p<0.001, ****p<0.0001).

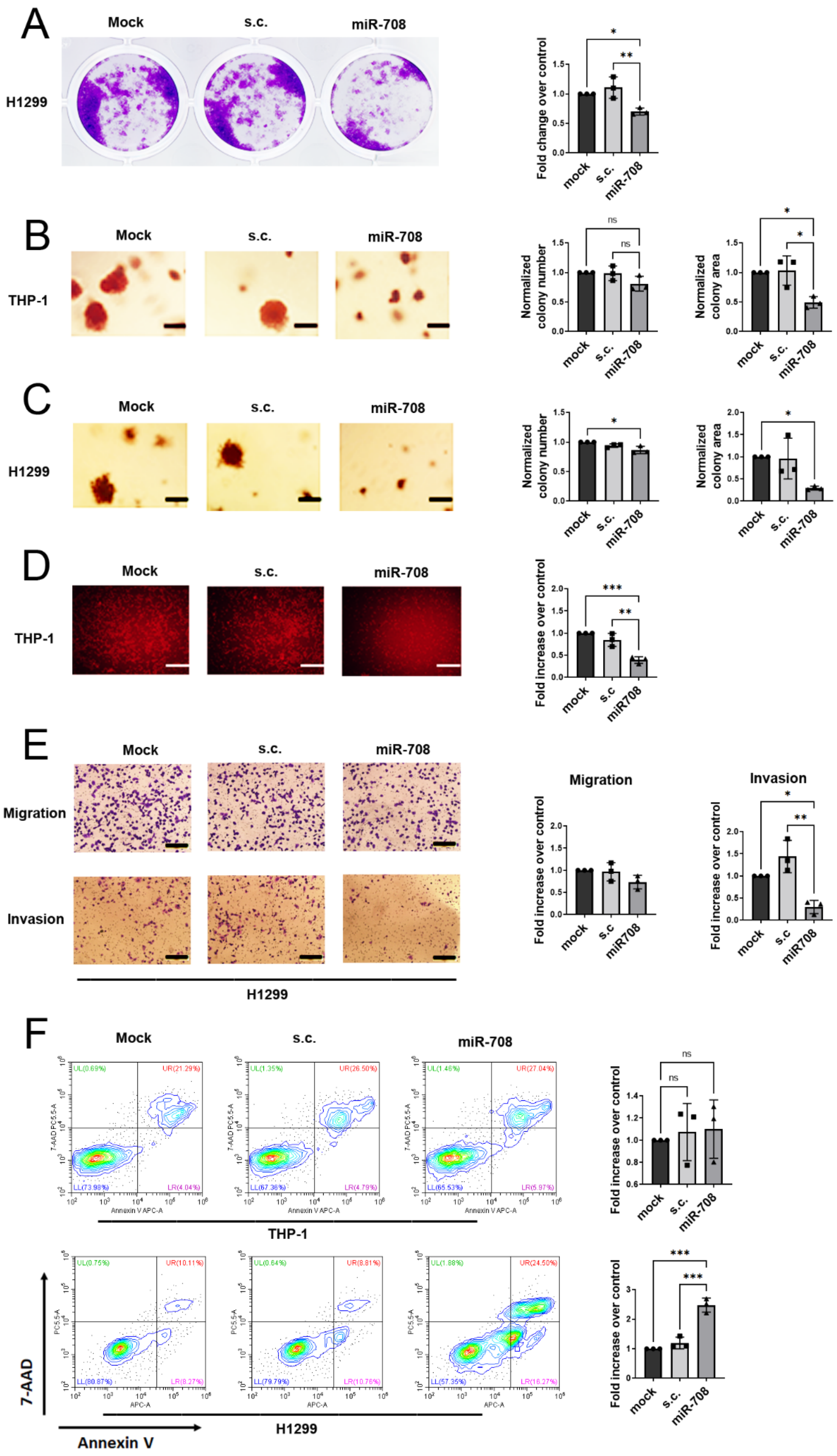
A
Mock
s.c.
miR-708
H1299
Fold change over control
mock
s.c.
miR-708
B
Mock
s.c.
miR-708
THP-1
Normalized colony number
Normalized colony area
ns
C
Mock
s.c.
miR-708
H1299
Normalized colony number
Normalized colony area
D
Mock
s.c.
miR-708
THP-1
Fold increase over control
mock
s.c
miR708
E
Mock
s.c.
miR-708
Migration
Invasion
H1299
Migration
Invasion
Fold increase over control
F
Mock
s.c.
miR-708
Annexin V APC-A
THP-1
Fold increase over control
ns
APC-A
H1299
7-AAD
Annexin V

Figure S3. MicroRNA-708 exerts tumor suppressive function in H1299 and THP-1 cells. (A) Clonogenic assay of H1299 cells transfected with mock, s.c., and pre-miR-708. Left: representative images of H1299 cells are shown. Right: histogram represents the fold change of mean ± SD (n=3 biological replicates) relative to mock. (B, C) Soft agar colony formation assay was performed in (B) THP-1, (C) H1299 cells expressing mock, s.c., miR-708. Left: the representative images of (B) THP-1 and (C) H1299 cells. Scale bars: 200 µm. Right: the number and size of colonies were quantified and expressed as fold increase compared to mock (n=3 biological replicates). (D) Chemotaxis assay of THP-1 cells transfected with mock, s.c., miR-708. Left: the representative images of THP-1 cells stained with red fluorescent dye. Scale bars: 200 µm. Right: the quantitative result of chemotaxis assay. Data are the means ± SD (n=3 biological replicates) relative to mock. (E) H1299 cells expressing mock, s.c., and miR-708 were subjected to transwell cell migration and invasion assay. Left: the representative images of migration and invasion. Scale bars: 200 µm. Right: the quantitative results of migration and invasion assay. Data are the means ± SD (n=3 biological replicates) relative to mock. (F) Apoptosis of THP-1 and H1299 cells overexpressing mock, s.c. and miR-708. Cells were incubated with 200 µM of H_2O_2 for 24h and then examined by Annexin V/7-AAD staining through flow cytometry analysis. The apoptotic rate was represented by the percentage of Annexin V positive cells and then normalized with mock. Data are fold changes ± SD (n=3 biological replicates) relative to mock. (A-F) One-way ANOVA followed by Tukey's post-hoc test was used for all statistical tests (*p<0.05, **p<0.01, ***p<0.001, ****p<0.0001).

Raw blot - Figure 1B

SK-MEL-2_20191009

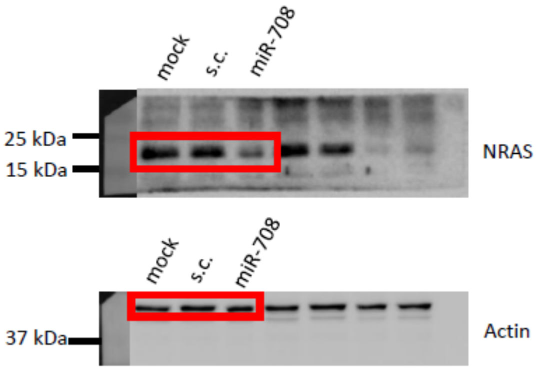

H1299_20210623

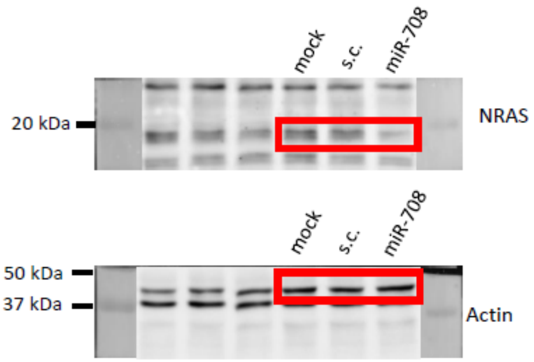

*Re-probed blot from stripped p-ERK blot

THP-1_20210127

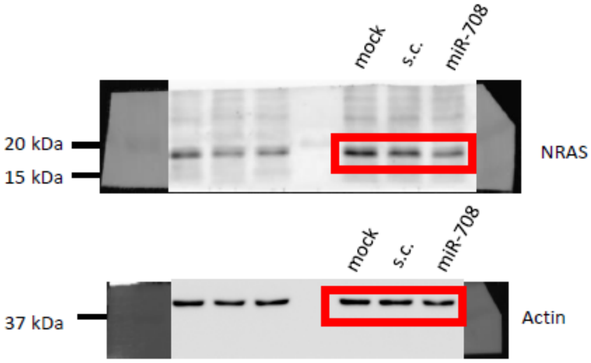

Raw blot - Figure 3A

SK-MEL-2_20191009

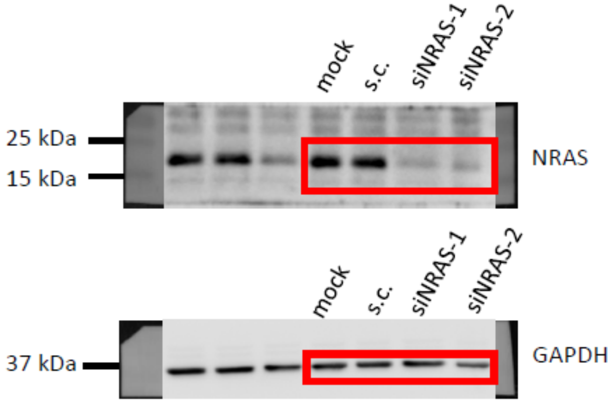

Raw blot - Figure 3F

SK-MEL-2_20191009

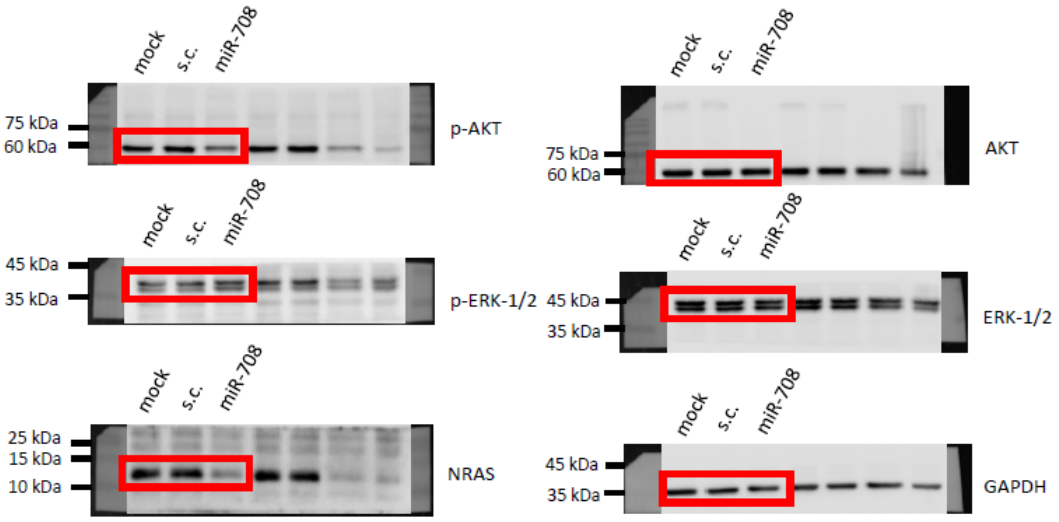

THP-1_20210112

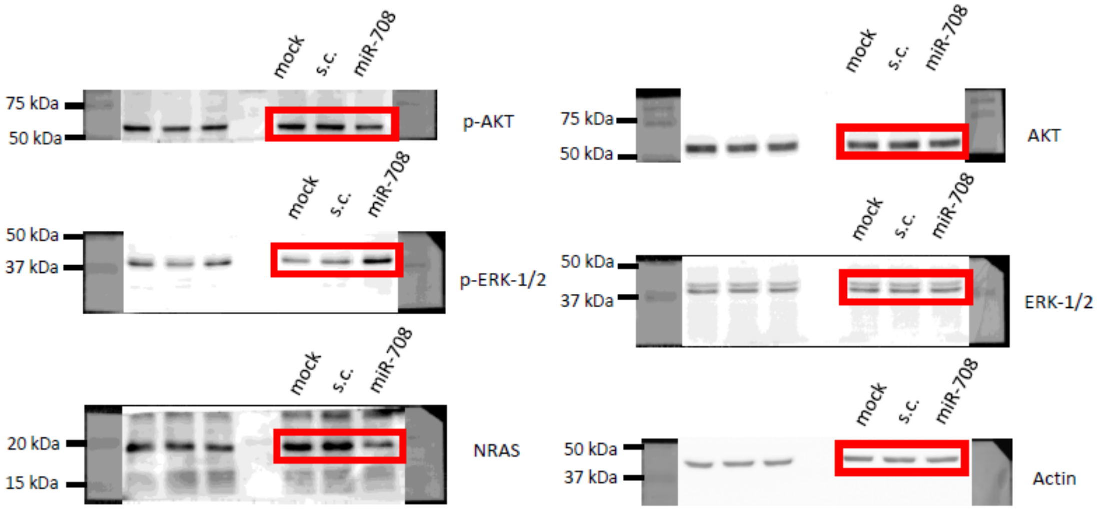

Raw blot - Figure 3F

H1299_20211215

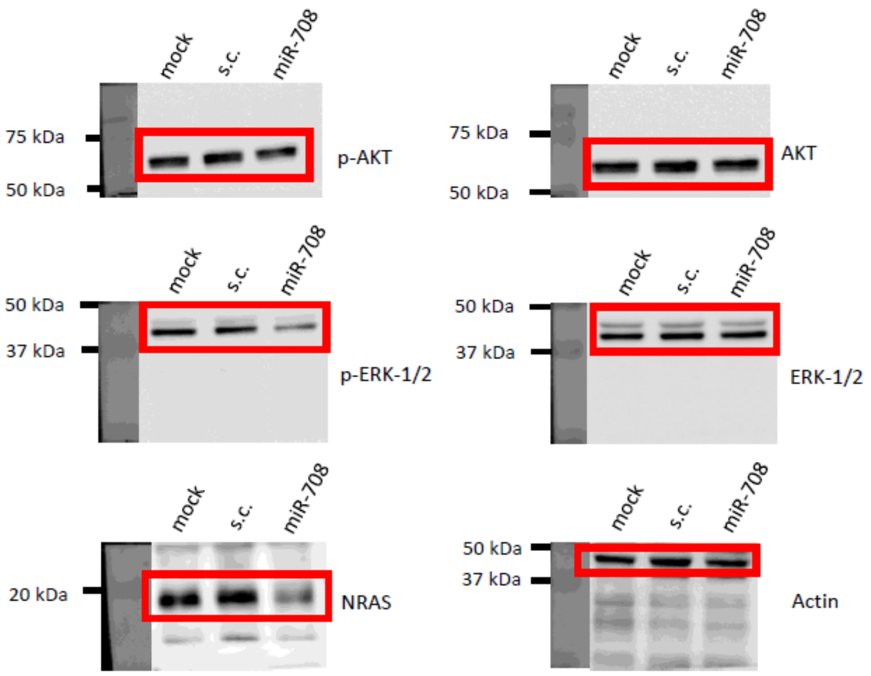

Figure 4A

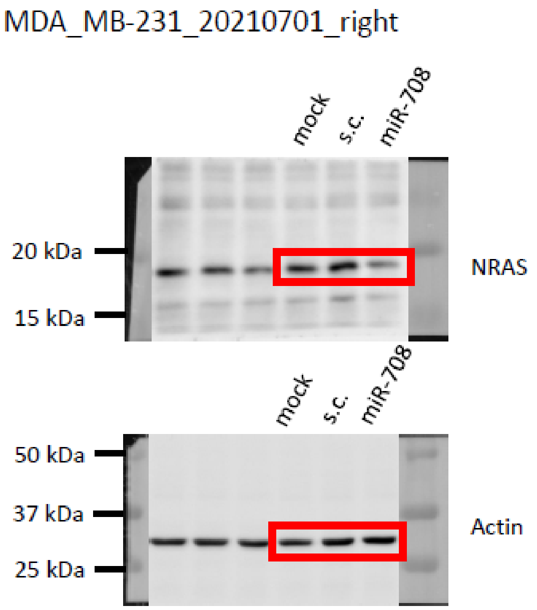

Figure 4C

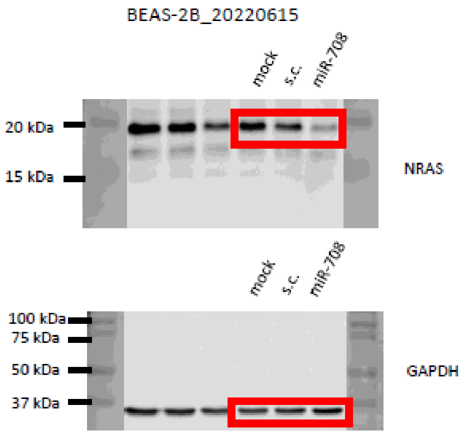

Figure S4. Uncropped images underlying of all blots.

Pre-miR-708	AACUGCCCUCAAGGAGCUUACAAUCUAGCUGGGGGUAA AUGACUUGCACAUGAACACAACUAGACUGUGAGCUUCUA GAGGGCAGGGA
Scramble control	Sense: UUCUCCGAACGUGUCACGUTT<br>Anti-sense: ACGUGACACGUUCGGAGAATT
siNRAS-1	Sense: UCCAGAACCACUUUGUAGATT<br>Anti-sense: UCUACAAAGUGGUUCUGGATT
siNRAS-2	Sense: ACCUGUUUGUUGGACAUACTT<br>Anti-sense: GUAUGUCCAACAACAAACAGGUTT
miR-708-RT primer	GTTGGCTCTGGTGCAGGGTCCGAGGTATTCGCACCAGAGC CAACCCCAGC
U6-RT primer	GTTGGCTCTGGTGCAGGGTCCGAGGTATTCGCACCAGAGC CAACAAAAATAT
miR-708 forward primer	CGGCGGAAGGAGCTTACAATCTA
U6 forward primer	TTCCTCCGCAAGGATGACACGC
Universal reverse primer	GTGCAGGGTCCGAGGT
NRAS forward primer	TGGAGCTTGAGGTTCTTGCT
NRAS reverse primer	TCGCCTGTCCTCATGTATTGG
Actin forward primer	CGGCATCGTCACCAACTG
Actin reverse primer	TCTCAAACATGATCTGGGTCATCT
pGL3-NRAS-3’UTR forward primer	ATATGAATTCCTTTCAAGCTGCACTGACAC
pGL3-NRAS-3’UTR reverse primer	ATATGGCCGGCCGCCAAGTGAGGAGGTAGTTATTC
NRAS-3’UTR-mut forward primer	TCTCAGAGAGATCGACCTGCTACTTCCCCA
NRAS-3’UTR-mut reverse primer	TCGATCTCTGTGAGACTGAACGCAGCAACAGG

Table S1. Primer and small RNA sequences.
