# Supplementary material for: MicroRNA-708 emerges as a potential candidate to target undruggable NRAS
Source: PLoS One. 2023 Apr 21;18(4):e0284744. doi: 10.1371/journal.pone.0284744 (PMC10120925; doi:10.1371/journal.pone.0284744)
Supplement: Attachment — Submitted filename: Response to reviewers-PLOS ONE-03-20-2023.pdf [file pone.0284744.s003.pdf]

Review Comments to the Author

Reviewer #1: The present manuscript describes a report using miRNA-708 that targets the NRAS gene to develop a miRNA-based management of NRAS mutation-driven cancers. They show that NRAS is a direct target of miRNA-708 which reduce NRAS protein levels in cell lines with NRAS mutation (melanoma, leukemia, and lung cancer), resulting in suppressed cell proliferation, anchorage independent growth, and promotion of reactive oxygen species-induced apoptosis. Its results also shown that the activities of NRAS-downstream effectors, PI3KAKT-mTOR or RAF-MEK-ERK signaling pathway, were affected in miR-708 over-expressing cells. Moreover, they shown that cell proliferation was not disturbed by miRNA-708 in cell lines carrying wild-type NRAS. They conclude that their results unveil the therapeutic potential of miRNA-708 in NRAS mutation-driven cancers as a precision medicine in cancer treatment.
In my opinion the study is well conducted and the results support the claimed conclusions. However, I believe that the manuscript can be improved resolving the following concerns.

Made clear in the whole manuscript when the symbols are referred to proteins or its genes…

Write properly the symbols of genes described (italics). Ej. RAS, NRAS and HRAS in the whole document when refers to gene names…

**Responses:** Thank you for the reminder. We have made changes to this. All the symbols that referred to the gene are italicized and non-italicized for protein.

The last sentence in Survival Correlation Analysis in Material & Methods section is incomplete (page 13, line 249).

**Responses:** Sorry for the mistake. The incomplete sentence is now removed from the section.

Fig 5 A, properly indicate the legend…

**Responses:** Thank you for the suggestion. We have reorganized the figure legend with a more detailed description.

Respect to the results of the miRNA-708 over-expression on the activities of NRAS-downstream effectors, PI3KAKT-mTOR or RAF-MEK-ERK signaling pathway, a paragraph with information about other miRNA-708 targets that could explain the results on the activity of the ERK in SK-MEL-2 and TPH-1 cell lines should be included.

**Responses:** Thank you for the suggestion. we have added a paragraph in the Discussion to discuss the possible causes of the differential activity between PI3K/AKT/mTOR or RAF/MEK/ERK by miR-708 in these different cell lines (Line 397-420).

The survival-miRNA-708 expression correlation study results should be included in the discussion section…

**Responses:** Thank you for the suggestion. we have added a paragraph in the Discussion to acknowledge the limitations of small sample sizes and potential biases (Line 439-450).

I recommend the review of the manuscript by a native English language speaker

**Responses:** Thanks for the suggestion and the revised manuscript was reviewed by an English language editing service. The editing certificate is shown below.

AJE

## Editing Certificate

This document certifies that the manuscript

**MicroRNA-708 emerges as a potential candidate to target undruggable NRAS**

prepared by the authors

**Jia Meng Pang, Po-Chen Chien, Ming-Chien Kao, Pei-Yun Chiu, Pin-Xu Chen, Yu-Ling Hsu, Chengyang Liu, Xiaowei Liang, and Kai-Ti Lin**

was edited for proper English language, grammar, punctuation, spelling, and overall style by one or more of the highly qualified native English speaking editors at AJE.

This certificate was issued on **March 10, 2023** and may be verified on the AJE website using the verification code **5BF6-8353-3C5A-54DA-09FA**.

AJE EDITING CERTIFIED

Neither the research content nor the authors' intentions were altered in any way during the editing process. Documents receiving this certification should be English-ready for publication; however, the author has the ability to accept or reject our suggestions and changes. To verify the final AJE edited version, please visit our verification page at aje.com/certificate. If you have any questions or concerns about this edited document, please contact AJE at support@aje.com.

AJE provides a range of editing, translation, and manuscript services for researchers and publishers around the world. For more information about our company, services, and partner discounts, please visit aje.com.

Reviewer #2: 1. Based on the experimental evidence provided by the authors, it is not clear whether NRAS is a direct or indirect target of mir-708. Is there any experimental evidence of NRAS and mir-708 interaction?

**Responses:** The experimental evidence showing the direct interaction between NRAS 3'UTR and miR-708 is in Figure 1D. We cloned NRAS 3'UTR into Luciferase reporter plasmid. Heterologous reporter assay (3'UTR of suspected target gene mRNA is cloned into reporter plasmid and tested for its response with that miRNA, with and without mutation on the predicted miRNA binding site) is the golden standard in the field to validate the direct interaction between miRNAs and target gene. Our data showed that miR-708 decreased the luciferase activity of reporter plasmid that carried 3' UTR of NRAS mRNA and mutations on the miR-708 potential binding sites abrogated the effect. This assay provided strong evidence to support that NRAS is a direct target of miR-708, and the binding site is as predicted.

2. The authors should have included another control cell line with normal NRAS

expression in order to draw the conclusion that the overexpression of miRNA-708 decreased the NRAS protein levels in the cell lines with NRAS mutation.

**Responses:** Thanks for the suggestion. In the revised manuscript, we included A549, a human lung adenocarcinoma cell line with $KRAS^{G12S}$ mutation and wild-type NRAS. Our data showed that miR-708 could effectively suppress NRAS expression without affecting A549 proliferation (Figure 4C, D), which is consistent with another KRAS mutant cancer cell line, MDA-MB-231 (Fig. 4A, B), and lung normal cell line, BEAS-2B (Fig. 4E, F). This supported our hypothesis that miR-708 can act as a potential targeted therapeutic molecule for NRAS-mutant cancers without affecting normal tissues.

3. Overexpression of mir-708 only has a considerable anti-tumour impact on SK-ME-2 cells compared to the other two cell lines. Not all of the metrics taken into consideration for THP-1 and H1299 cells show a significant anti-tumour impact. In the result section (lines 308-310), the assertion drawn from the experiments does not indicate the same.

**Responses:** Thanks for pointing out the issue with data interpretation. We have modified our wording into more conservative. We think different antitumor effects observed in different cell lines are reasonable since their RAS signaling was differently regulated. The discrepancies in mechanistic level might exploit an opportunity for combinatorial therapies to enhance the antitumor effect.

4. The lack of a robust anti-tumour effect from NRAS knockdown in SK-ME-2 raises the question of whether it is a potent therapeutic target.

**Responses:** According to our data, miR-708-mediated and siRNA-mediated NRAS silencing showed comparable inhibition in clonogenic proliferation, soft agar colony forming ability, invasion, and ROS-induced apoptosis and only exhibited a difference in migration abrogation. Since NRAS knockdown did exert pronounce antitumor effects on almost every ability that defines the aggressiveness of cancer progression (Figure 2, 3), we believe that it is a potent therapeutic target. More importantly, depletion of NRAS by miR-708 does not inhibit cell proliferation rate in cells carrying wild-type NRAS (Figure 4), suggesting that using miR-708 to target NRAS mutation-driven cancer is a safe and less toxic approach for cancer patients. Taking the high specificity and low

toxicity into consideration, we believe miR-708 is still a strong candidate for future drug development.

5. What could be the plausible explanation for the differential response of mir-708 overexpression towards downstream effectors of NRAS?

**Responses:** To discuss this issue and to give a plausible explanation, we have added a paragraph in Discussion to discuss the causes of the differential activity between PI3K/AKT/mTOR or RAF/MEK/ERK by miR-708 in different cell lines (Line 397-420).

6. In the Kaplan-Meier plot, the P value is quite high for the data to be considered significant. Thereby the assertions drawn may not hold true.

**Responses:** We agreed that due to the limited sample sizes, the significance may contain statistical bias. Therefore, we have added a paragraph in the Discussion to acknowledge the limitations of small sample sizes and potential biases (Line 439-450).
